# Three-Week Inpatient Treatment of Obsessive-Compulsive Disorder: A 6-Month Follow-Up Study

**DOI:** 10.3389/fpsyg.2018.00620

**Published:** 2018-04-30

**Authors:** Torun Grøtte, Bjarne Hansen, Svein Haseth, Patrick A. Vogel, Ismail C. Guzey, Stian Solem

**Affiliations:** ^1^Department of Psychology, Norwegian University of Science and Technology, Trondheim, Norway; ^2^Nidaros DPS, St. Olav’s University Hospital, Trondheim, Norway; ^3^Department of Psychiatry, Haukeland University Hospital, Bergen, Norway; ^4^Faculty of Psychology, University of Bergen, Bergen, Norway; ^5^Division of Psychiatry, Department of Research and Development, St. Olav’s University Hospital, Trondheim, Norway; ^6^Department of Neuroscience, Norwegian University of Science and Technology, Trondheim, Norway

**Keywords:** obsessive-compulsive disorder (OCD), inpatient treatment, follow-up, effectiveness, exposure with response prevention (ERP), cognitive-behavioral therapy (CBT)

## Abstract

**Background:** Specialized inpatient or residential treatment might be an alternative treatment approach for patients with obsessive-compulsive disorder (OCD) that do not respond satisfactorily to the standard outpatient treatment formats.

**Method:** The aim of this open trial was to investigate the 6-month effectiveness of a 3-week inpatient treatment of OCD, where exposure with response prevention (ERP) was the main treatment intervention. The sample consisted of 187 adult patients with OCD, all with previous treatment attempts for OCD.

**Results:** The sample showed significant reductions in symptoms of OCD and depression. The effect sizes were large for obsessive-compulsive symptoms and moderate to large for depressive symptoms. At discharge, 79.7% of the intent-to-treat (ITT) group were classified as treatment responders (≥35% reduction in Y-BOCS scores). However, some participants experienced relapse, as 61.5% of the ITT group were classified as treatment responders at 6-month follow-up. Antidepressant use appeared not to influence the outcome. Only pre-treatment levels of obsessive-compulsive symptoms emerged as a significant predictor of relapse.

**Conclusion:** The 3-week inpatient programme produced similar treatment effects as previous inpatient and residential studies of longer duration (2 – 3 months). The results suggest that patients with severe OCD can be treated efficiently using this brief inpatient format. However, better relapse prevention interventions are needed.

## Introduction

The efficacy of cognitive-behavioral therapy (CBT) in treatment of obsessive-compulsive disorder (OCD) has been confirmed in numerous studies and meta-analyses (e.g., Eddy et al., 2004; Öst et al., 2015; Skapinakis et al., 2016). According to the National Institute for Health and Clinical Excellence guidelines’ (National Institute for Health and Clinical Excellence [NICE], 2005), outpatient CBT including exposure and response prevention (ERP) should be offered to all OCD patients. Among treatment completers, significant improvement in obsessive-compulsive symptoms is achieved by two-thirds of the patients, which corresponds to uncontrolled effect sizes around 1.5 at post-treatment (Eddy et al., 2004). In intention-to-treat samples including treatment dropouts, about half of the patients experience 25–50% reduction in their obsessive-compulsive symptoms (Eddy et al., 2004). This leaves a subset of OCD patients failing to achieve satisfactory response to CBT, and many experience residual symptoms or relapse during follow-up (Simpson et al., 2005; Abramowitz, 2006).

In cases where OCD patients have not responded satisfactorily to first-line standard outpatient treatments such as CBT, specialized inpatient or residential treatment might be an alternative treatment approach (National Institute for Health and Clinical Excellence [NICE], 2005). A review of inpatient and residential treatment programs using CBT for OCD (Veale et al., 2016b) showed that patients with severe or treatment refractory OCD can make significant improvements with this treatment format. However, there is a lack of knowledge regarding its long-term benefits, especially regarding treatments of shorter duration. Consequently, the primary aim of the current study was to explore the 6-month effectiveness of a brief (3 weeks) inpatient treatment format.

An inpatient setting has therapy and support staff available both day and night, whereas a residential setting has nursing staff during the day only. According to the National Institute for Health and Clinical Excellence [NICE] (2005) guidelines’, inpatient or residential treatment may be beneficial when there is extreme distress or functional impairment, risk to life, severe self-neglect, or where a patient has comorbid disorders that make outpatient treatment more complex. Inpatient services may also be required when the response to previous OCD treatment was poor and the patient needs more intensive CBT or more assisted exposure than what is possible to deliver in an outpatient setting. However, a major drawback is the cost of treatment, as well as the different context in which the obsessive-compulsive symptoms naturally occur. Compared to an outpatient setting, an inpatient or residential treatment may give fewer opportunities to consolidate and generalize the learning to the patients’ home environment (Craske et al., 2008).

Several naturalistic studies have investigated the effectiveness of specialized inpatient or residential treatment for OCD, and the results are promising (e.g., Stewart et al., 2005; Boschen et al., 2008; Langner et al., 2009; Adams et al., 2012; Björgvinsson et al., 2013; Dowling et al., 2016; Veale et al., 2016a). Two of the largest studies of inpatient and residential OCD treatment to date are conducted by Stewart et al. (2005) and Veale et al. (2016a).

Stewart et al. (2005) investigated the effectiveness of an American intensive residential treatment unit with a mean treatment duration of 66 days (*N* = 403). Results indicated a mean improvement in OCD symptoms of 30%, as Yale-Brown Obsessive-Compulsive Scale (Y-BOCS) scores decreased from pre-treatment (*M* = 26.6, *SD* = 18.6) to post-treatment (*M* = 18.6, *SD* = 7.2). Depressive symptoms also improved significantly. Follow-up scores were not reported, but in a second study using a different OCD sample from the same residential treatment unit, Stewart et al. (2009) found treatment gains to be stable 6 months after discharge. However, one limitation of the follow-up study was low sample size (*N* = 36 at 6 months).

Veale et al. (2016a) described treatment outcomes from a British residential unit, where one of the aims was to compare an intensive treatment program of 2 weeks (*N* = 54) to a “standard” treatment program of 12 weeks (*N* = 418). Results indicated significant decreases from pre- to post-treatment in both groups. In the standard treatment program, Y-BOCS scores decreased from a mean of 30.8 (*SD* = 6.0) to an average discharge score of 18.6 (*SD* = 7.8). In the intensive treatment program, Y-BOCS scores decreased from 30.1 (*SD* = 5.5) to 20.8 (*SD* = 7.5). There was no significant difference in treatment outcome between the two programs. However, Y-BOCS follow-up scores were only reported on the 12-week program, with 6 – 12 month follow-up scores showing a slight deterioration in outcome compared to discharge (*M* = 22.6, *SD* = 7.9).

As previously mentioned, Veale et al. (2016b) conducted a systematic review of inpatient and residential treatment programs using CBT for OCD. The analysis included 19 studies with a total of 2306 participants at admission and 2202 participants at discharge. The average length of stay was 10.4 weeks (range: 5.0 – 19.3 weeks), whereas the average mean Y-BOCS at pre-treatment was 27.6^1^ (range: 24.1 – 34.7). The meta-analysis showed a substantial amount of heterogeneity in the estimate of treatment effect size, which Veale et al. (2016b) partly explained by the wide nature of the treatment programs offered, as well as variations in admission criteria. Nonetheless, a large treatment effect with Hedges *g* of 1.87 and a mean improvement of 10.7 (95% CI: 9.8 – 11.5) points from admission to discharge on the Y-BOCS were found. Accordingly, Veale et al. (2016b) concluded that inpatient and residential treatment is an encouraging option for those with severe or treatment refractory OCD. As few studies have evaluated the long-term effectiveness of their inpatient or residential treatment, Veale et al. (2016b) did not calculate mean change in Y-BOCS scores from admission to follow-up. However, the results of the few studies that have reported long-term outcomes are inconsistent, with some studies reporting the gains to be stable (e.g., Kordon et al., 2005; Rufer et al., 2005; Stewart et al., 2009), whereas some found a slight deterioration (McKenzie and Marks, 2003; Veale et al., 2016a). Research on predictors of long-term outcome is also scarce. However, Stewart et al. (2009) found that patients who relapsed at 6-month follow-up were significantly more likely to be living alone and *less* likely to have comorbid illnesses.

In general, the main finding of previous naturalistic research on the effectiveness of inpatient and residential treatment for OCD indicates that inpatient services may be a viable treatment option for patients who do not respond satisfactorily to outpatient treatment. However, there is a lack of studies with follow-up data, and the studies that do report follow-up are often limited by low sample size. Furthermore, most of the previous studies (84.2% of the studies included in the meta-analysis by Veale et al., 2016b) report inpatient or residential treatments with duration of 2–3 months, and there are only a few studies (e.g., Veale et al., 2016a) that have investigated the effectiveness of a brief inpatient or residential format. From both cost-effectiveness and a clinical perspective, it is important to explore whether a briefer inpatient or residential treatment format could produce similar results as treatment formats of longer duration, both at discharge and at a longer term.

Consequently, the primary aim of this study was to explore the 6-month effectiveness of a brief psychological (no changes in medication) inpatient treatment format with duration of 3 weeks. In light of recent research on intensive treatment formats (e.g., Jónsson et al., 2015), it was hypothesized that obsessive-compulsive symptoms would show a significant reduction from admission to discharge, with some deterioration between discharge and 6-month follow-up. As a secondary outcome measure, the outcome pattern of depressive symptoms was examined; the hypothesis being that depressive symptoms would show a similar pattern as obsessive-compulsive symptoms. Third, since the majority of previous studies of inpatient and residential treatment of OCD see pharmacotherapy as an integral component of their treatment (e.g., Rufer et al., 2005; Stewart et al., 2005; Adams et al., 2012), our last aim was to explore the influence of antidepressant medication on treatment outcome.

## Materials and Methods

### Participants and Procedure

The sample consisted of 187 consecutively admitted patients with OCD who were first-time admissions to a 3-week inpatient treatment in a specialized anxiety unit in Norway. Out of these, 166 were treatment completers and 21 (11.2%) were dropouts. Patients were included in this study if they met DSM-IV criteria for OCD according to the Anxiety Disorder Interview Schedule (ADIS-IV, Brown et al., 1994), and if OCD was considered the principal diagnosis. The primary reasons for admission to the specialized inpatient treatment were inadequate response to prior outpatient treatment and/or presence of severe OCD.

**FIGURE 1:**
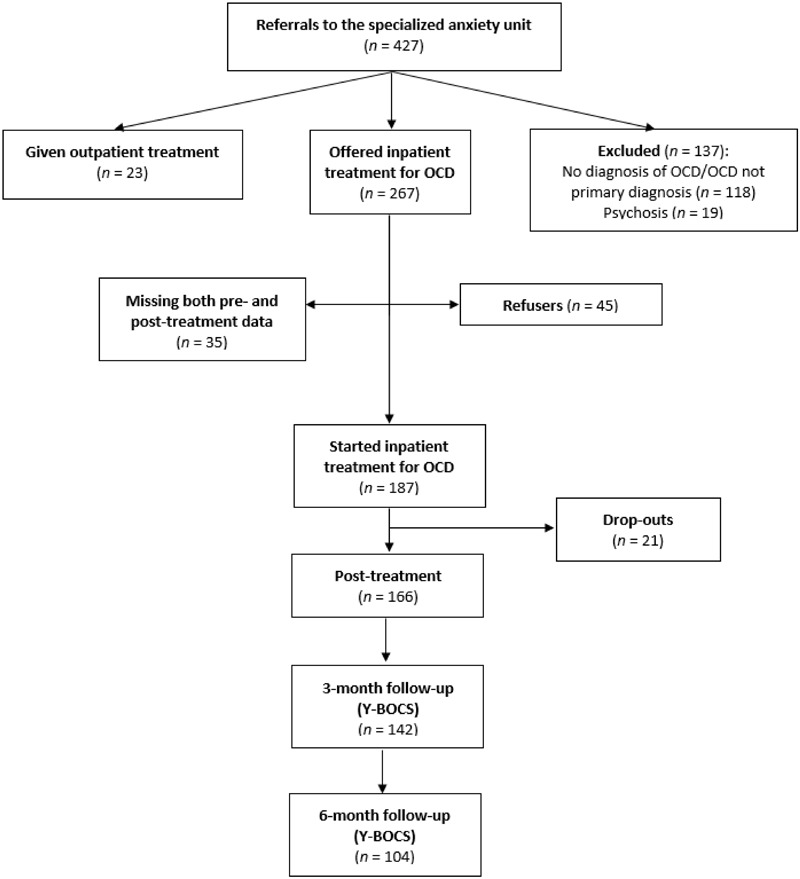
Participant flow chart. OCD, Obsessive-compulsive disorder; Y-BOCS, Yale-Brown Obsessive-Compulsive Scale.

As shown in the participant flow diagram (**Figure 1**), 427 patients were originally assessed for inclusion in this study. Exclusion criteria were as follows: no diagnosis of OCD/OCD not primary diagnosis (*n* = 118); psychosis (*n* = 19); patients assessed as eligible for outpatient treatment rather than inpatient treatment (*n* = 23). Forty-five (19.4%) patients were offered inpatient treatment, but refused. Furthermore, 35 OCD patients treated at the clinic were not included in the analysis due to missing both pre- and post-treatment data. The data was collected through paper and pencil and internet administration. All outcome measures were part of the standard quality control instruments of the health services offered at the inpatient unit. The medical quality registry was approved by the local Data Protection Official for Research (Reference Number: 12/5755) and the Regional Committee for Medical and Health Research Ethics (Reference Number: 2010/2883). All subjects gave written informed consent in accordance with the Declaration of Helsinki.

### Treatment

The inpatient treatment program for OCD was organized into separate phases for assessment and treatment. At their first arrival at the unit, the OCD patients went through a thorough diagnostic evaluation and assessment of suitability for treatment. This assessment had a duration of 3 to 5 days and was performed by the therapists at the inpatient unit. In addition to the differential diagnostic evaluation where ADIS-IV (Brown et al., 1994) and several self-report questionnaires (e.g., The Obsessive-Compulsive Inventory-Revised; Foa et al., 2002) were used, a detailed summary of the patients’ obsessions and compulsions was made. This was later used as a basis for formulation of the anxiety hierarchy and designation of exposure exercises.

Another important ingredient in the assessment phase was psychoeducation concerning anxiety and OCD, as well as providing a rationale for ERP. The rationale given was not based on habituation or disconfirmation of cognitive beliefs, but with a main focus on how the exposure exercises should be conducted. The patients were informed that obsessional thoughts were normal and without risk, and that OCD was developed and maintained by strategies with an aim of controlling obsessional thoughts. An important aim of treatment was therefore to teach the patients to confront their obsessional thoughts and feelings in the opposite way, i.e., seek out anxiety and discomfort rather than trying to reduce and/or control it. For learning and motivational purposes, the patients also tried out a couple of exposure exercises. In the end of the assessment stay, a 30-min motivational interview regarding the patients’ treatment motivation was conducted.

Due to waiting lists, the average time between assessment and treatment start was 3 months. The treatment phase had 3 weeks duration with behavioral elements (ERP) as the main ingredient. Pharmacotherapy in the form of antidepressant medication was allowed if the dose was maintained unchanged during the 3-week treatment phase, whereas use of benzodiazepines was not permitted. A minority of patients received cognitive (e.g., targeting dysfunctional beliefs like perfectionism and intolerance of uncertainty) or metacognitive (e.g., challenging thought-fusion beliefs and beliefs about rituals; Wells, 2009) interventions in addition to ERP. However, as the inpatient treatment program was not strictly manualized, there is no consistent information about the relative contribution of cognitive and metacognitive elements added to ERP.

As previously mentioned, the main treatment intervention was ERP. Patients performed a minimum of three exposure exercises per day; one accompanied by personnel, one partially assisted, and one without assistance. However, most patients conducted several exposure exercises daily, as full response prevention was an important aim from day one. An exposure exercise could last anywhere from 5 min to 1 h, depending on the issues and needs of the patient. To ensure that the exposure exercises were carried out in the right way (i.e., chasing anxiety and discomfort rather than trying to reduce and/or control it), the patients filled out behavioral experiment worksheets three times daily. Prior to exposure training, the patients were to describe the exposure exercise, what they feared would happen, as well as which rituals they normally would have used to reduce anxiety. Subsequent to exposure training, they evaluated the exercise by answering questions regarding anxiety level (0–100), degree of belief that anxiety symptoms were dangerous (0–100), degree of attempts to reduce/control anxiety and discomfort (0–100), as well as encouraging the patient to think of improvements (e.g., “What could be done to make the exposure exercise better?”). If the patients ritualized during an exposure session, they had to re-expose themselves to the trigger, as well as filling out a worksheet to evaluate what went wrong, what could be done to avoid ritualization in the future etc.

The patients were to reach the top of their exposure hierarchy within the first 1.5 weeks. Thereafter, during the second weekend in the treatment phase, the patients went home on leave to practice ERP, with an aim of generalizing and implementing learning. The exposure tasks that had been experienced as the most difficult during their leave home became the main focus of ERP the third and last week of treatment. The patients’ therapy progress was closely monitored by the staff, with multidisciplinary team meetings 3 days a week. The behavioral experiment worksheets, registration of deviations from response prevention, as well as check lists for the staff’s tasks during all phases of treatment, were also elements that were meant to ensure treatment structure and adherence to treatment.

Relapse prevention interventions, such as the formulation of an “old and new plan,” were also an important ingredient in the third week of treatment. The “old and new plan” is an overview of how the patients handled their obsessive-compulsive symptoms previously (e.g., regarding thinking style, behavior, and focus of attention), as well as what they had learnt regarding overcoming obsessive-compulsive symptoms during treatment. Also, relevant exposure exercises in their home environment were planned. The patients were offered short phone calls with therapists or milieu personnel up to 3 weeks after discharge, where they could repeat treatment rationale and evaluate exposure plans. The frequency of these phone calls varied depending on the patients’ needs.

Treatment completers were offered an additional 3-day stay at the inpatient clinic both at 3 and 6 months after discharge, with an aim of relapse prevention. In this period, interventions from the treatment phase were repeated. Among treatment completers, the rates of attendance to the follow-up stays were as follows: 77.2% at 3-month follow-up and 63.1% at 6-month follow-up. Patients who did not attend the 3-day follow-up stays were attempted to be reached by telephone. Therefore, 30 (10.4%) of Y-BOCS’ follow-up interviews were done by telephone.

### Therapists

The treatment was provided by a multidisciplinary team consisting of therapists and various personnel, including nurses, psychiatric nurses, social workers, and students. One therapist was assigned as a case supervisor and conducted regular individual therapy sessions, but the entire staff was involved in activities in the milieu, such as giving emotional and motivational support, as well as conducting exposure exercises. A total of 18 therapists were involved during this study’s recruitment period, and all were trained in diagnosis and treatment of OCD (i.e., ERP). Thirteen therapists had completed a 2-year course in cognitive behavioral therapy and/or metacognitive therapy. There was ongoing supervision by experienced ERP-therapists.

### Measures

The inventories were answered before assessment, before treatment start, at post-treatment, before 3-month follow-up, and before 6-month follow-up. To examine changes in symptoms of OCD and depression, the following measures were used:

#### The Yale-Brown Obsessive Compulsive Scale

The Yale-Brown Obsessive Compulsive Scale (Goodman et al., 1989) and the Yale-Brown Obsessive Compulsive Scale Self-Report (Y-BOCS-SR, Baer et al., 1993) were the primary outcome measures of this study, as they measure the severity of obsessive-compulsive symptoms. The respondents rated five aspects of both obsessions and compulsions: frequency, interference, distress, resistance, and control. A 5-point Likert scale ranging from 0 (*none*) to 4 (*extreme*) was used to rate the responses, where the range of severity was characterized as follows: 8 – 15 is considered as mild severity, 16 – 23 as moderate, 24 – 31 as severe, and 32 – 40 as extreme. The psychometrics of the Y-BOCS is well established (e.g., Steketee et al., 1996; Grabill et al., 2008). In our study, the interview version was used with the first 122 (65.2%) recruited participants. The self-report Y-BOCS was used with the last 65 (34.8%) participants when the inpatient unit switched to electronic assessments. However, there is a strong correlation between the two versions of Y-BOCS (Steketee et al., 1996). In this study, the Cronbach’s alpha coefficient measured at baseline was 0.80.

#### The Obsessive-Compulsive Inventory-Revised

Obsessive-Compulsive Inventory-Revised (OCI-R, Foa et al., 2002) is a self-report form that measures severity of obsessive-compulsive symptoms. The OCI-R consists of 18 items reflecting six subscales known as washing, checking, ordering, obsessions, hoarding, and mental neutralization. A 5-point Likert scale from 0 (*not at all*) to 4 (*extremely*) was used to rate the responses, where higher scores indicate more severe obsessive-compulsive symptoms (Foa et al., 2002). The OCI-R has demonstrated good reliability, and good convergent and discriminant validity (e.g., Foa et al., 2002; Abramowitz and Deacon, 2006). The psychometric properties are also validated in a Norwegian sample (Solem et al., 2010). In this study, the Cronbach’s alpha coefficient for the total scale at baseline was 0.83.

#### The Beck Depression Inventory

Beck Depression Inventory (BDI; Beck et al., 1961) is a widely used measure of severity of depressive symptoms. It is a self-report form with 21 items, where each symptom is rated on a 4-point Likert scale. The range of severity is characterized as follows: 10 – 14 is considered as mild severity, 15 – 24 as moderate, 25 – 63 as severe. The BDI is shown to be a highly reliable and valid measure of depression severity. In Beck et al. (1988) review of its psychometric properties, the internal consistency was 0.87, and test–retest reliability was greater than 0.60. Due to a switch into electronic assessment, the first 122 patients enrolled in our study answered the BDI, whereas the last 65 respondents answered the newer version, BDI-II (Beck et al., 1996). However, previous research has found the BDI and BDI-II to be strongly correlated (Dozois et al., 1998). The Cronbach’s alpha value was 0.89 at baseline.

### Data Analyses

First, possible differences in Y-BOCS, OCI-R, and BDI scores due to differences in administration mode (paper and pencil versus internet administration) and variability in the measures used (Y-BOCS versus Y-BOCS-SR, BDI versus BDI-II) were explored through independent *t*-tests with Bonferroni correction. Due to three comparisons, the critical alpha level was set at 0.02.

Second, to explore whether the inpatient group actually was different from an outpatient group, Pearson’s chi-square tests and independent *t*-tests were used to contrast the final sample’s baseline demographic and diagnostic information with characteristics of the OCD clinic’s outpatient sample from the same period of time. Furthermore, baseline demographic and diagnostic characteristics of study dropouts versus treatment completers were compared using the same statistics.

Not all measurements were available for every patient at follow-up. Among treatment completers (*n* = 166), number of respondents at 3-month follow up was as follows: Y-BOCS: *n* = 142; OCI-R: *n =* 139; BDI: *n* = 139. Among treatment completers at 6-month follow-up, number of respondents was 104 for Y-BOCS, 87 for OCI-R, and 86 for BDI. A non-significant Little’s MCAR test revealed that the data was missing completely at random: *χ^2^*(127) = 149.81, *p* = 0.082 (Little, 1988). Expectation maximization (EM) analysis was used to estimate the means and standard deviations for missing data at follow-up among the treatment completers. For each of the three scales, the available items from each wave of data were used in the calculations, i.e., the available items from assessment, pre-treatment, post-treatment, 3-month follow-up, and 6-month follow-up. For those with premature discharges (dropouts: *n* = 21), the last completed assessment was used in the analysis (via a last-observation-carried-forward-approach). Thus, an intention-to-treat (ITT) methodology was used in this study.

Treatment outcome data were analyzed using one-way repeated measures analysis of variance (ANOVA). No influential outliers were found, as measured by Cook’s distance. However, Mauchly’s test of sphericity indicated that the assumption of sphericity had been violated in the outcome variables: Y-BOCS: *χ^2^*(9) = 242.5, *p* = 0.001; OCI-R: *χ^2^*(9) = 281.7, *p* = 0.001; BDI: *χ^2^*(9) = 234.4, *p* = 0.001. Therefore, Greenhouse–Geisser corrected tests were reported. Effect sizes were reported as partial eta squared ($\eta_{p}^{2}$), where values of 0.01, 0.06, and 0.14 are considered to reflect small, medium, and large effects, respectively (Cohen, 1988).

As an additional measure of treatment effectiveness, the within-subject effect-sizes for changes in obsessive-compulsive and depressive symptoms were calculated with Cohens *d*, correcting for related means by using the equation by Morris and DeShon (2002). An effect size of 0.20 – 0.49 is considered small, 0.50 – 0.79 as moderate, and ≥ 0.80 as large.

To examine whether changes in obsessive-compulsive symptoms were clinically meaningful, clinically significant change analyses were carried out on Y-BOCS and OCI-R scores at post-treatment and 6-month follow-up. The international consensus criteria for Y-BOCS (Mataix-Cols et al., 2016) were chosen as the primary clinically significant change estimate, where *treatment response* is operationalized as a ≥ 35% reduction in Y-BOCS scores and a *partial treatment response* as a ≥ 25% but < 35% reduction. *Remission* is defined as having a Y-BOCS score ≤ 12.

Since inconsistencies in how treatment response are defined in clinical trials have been shown to lead to different estimates of treatment efficacy and relapse rates (Simpson et al., 2005), Fisher and Wells’ (2005) criteria were included as an additional clinical significance change measure on Y-BOCS scores. Thus, the proportion of patients who (1) *recover*, (2) make *statistically reliable improvement*, and (3) remain *unchanged* were calculated. To meet criteria of recovery, the patients needed a score of 14 points or less, as well as a reliable change index (RCI) of minimum 10 points change following treatment. To achieve “statistically reliable improvement,” only the RCI criteria of minimum 10 points change applied. The proportion of patients that had a post-treatment and 6-month follow-up score at or below the cut-off (≤14) was also calculated.

In the clinically significant change analysis on OCI-R scores, the same classification of recovery, statistically reliable improvement, and no change, was used. Similar criteria as used by Solem et al. (2009) were applied, with a cut-off score of 21 and RCI of 12.

The potential influence of antidepressant use on treatment outcome was investigated using independent *t*-tests, where the Y-BOCS post-treatment and 6-month scores of antidepressant users versus non-users were contrasted. In addition, the impact of antidepressant dose was explored using the World Health Organization’s (World Health Organization Collaborating Centre for Drug Statistics Methodology [WHO], 2015) “defined daily doses” (DDD) methodology. Each patients’ antidepressant dose was converted into a DDD-score, where all DDD’s were based on the World Health Organization Collaborating Centre for Drug Statistics Methodology [WHO] (2015) guidelines. To explore the relationship between dose and Y-BOCS treatment outcome, correlational analyses of Y-BOCS post-treatment and 6-month scores and DDD-scores were conducted.

Lastly, a logistic regression analysis was used to explore predictors of relapse at 6-month follow-up. Relapse was defined using Mataix-Cols et al.’s (2016) criteria; the patient initially classified as being in remission (i.e., a Y-BOCS score of 12 or lower), but no longer met this criteria at follow-up. The patients’ relapse status (relapse versus remained in remission) was entered as the dependent variable, whereas gender, age, marital status (single vs. married/cohabitant), pre-treatment Y-BOCS and BDI scores, and attendance to the follow-up stay 3 months after discharge were entered as predictor variables. Multicollinearity did not appear as a problem with any of the predictor variables, with VIF ranging from 1.03 – 1.28 and tolerance from 0.78 to 0.98. No influential observations were found (measured by Cook’s distance, leverage, and the standardized residuals).

## Results

### Preliminary Analyses

The data was collected through both paper and pencil (*n* = 122 at baseline) and internet administration (*n* = 65 at baseline). Independent t-tests showed no significant differences between the two administration modes at baseline, neither in Y-BOCS scores, *t*(185) = 0.48, *p* = 0.634, OCI-R scores, *t*(185) = -0.05, *p* = 0.957, nor BDI scores, *t*(93.5) = -1.42, *p* = 0.160.

### Participant Characteristics

A description of the final sample’s baseline demographic and diagnostic information is provided in **Table 1**. Their characteristics are contrasted with characteristics of the same clinic’s outpatient OCD sample from the same time period. Significant differences between the two samples were found, indicating higher severity of obsessive-compulsive symptoms and life impairment in the inpatient sample. The inpatients were older, reported more severe obsessive-compulsive symptoms, and lower rates of employment/student status. Also, the inpatients had a more extensive treatment history and used more antidepressant medication.

**Table 1:** Comparison of demographic and diagnostic characteristics among inpatients and outpatients with OCD from the same anxiety clinic.

	Inpatients *(N = 187)*	Outpatients *(N = 91)*		
Characteristic	*%*	*%*	*p*	*Phi*
Female gender	63.6	64.8	0.894	-0.01
Employed/student	48.9	66.7	**0.004**	-0.18
Married/cohabitant	34.8	40.7	0.355	0.06
Using antidepressants	54.0	26.7	**0.001**	-0.26
Previous outpatient treatment	99.5	81.3	**0.001**	-0.35
Previous inpatient treatment	36.8	17.6	**0.001**	-0.20

	***M (SD)***	***M (SD)***	***t***	***p***

Age	34.24 (11.2)	29.90 (10.1)	3.13	**0.002**
Y-BOCS	26.03 (4.8)	22.02 (4.3)	6.70	**0.001**
OCI-R	30.22 (12.6)	24.22 (12.0)	3.73	**0.001**
BDI	19.29 (9.9)	20.42 (12.1)	-0.76	0.447

*Significant *p*-values are in boldface. OCD, Obsessive-compulsive disorder; Y-BOCS, Yale-Brown Obsessive-Compulsive Scale; OCI-R, Obsessive-Compulsive Inventory-Revised; BDI, Beck Depression Inventory.*

**FIGURE 2:**
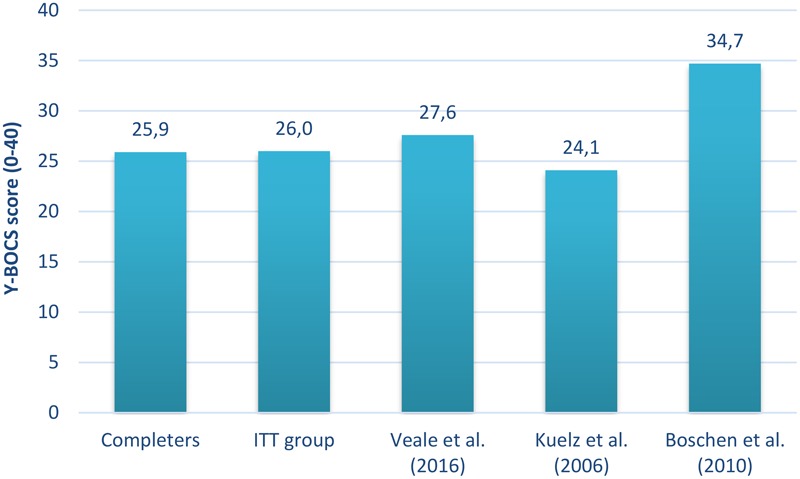
Comparison of pre-treatment Y-BOCS between studies on inpatient and residential treatment for OCD. The mean pre-treatment Y-BOCS scores of treatment completers (*n* = 166) and the ITT group (*n* = 187) from the current study were contrasted with the mean pre-treatment Y-BOCS from the 19 inpatient and residential studies included in Veale et al. (2016b), as well as the studies with the lowest and highest mean Y-BOCS pre-treatment value in Veale et al. (2016b). Y-BOCS, Yale-Brown Obsessive-Compulsive Scale; ITT group, intention-to-treat sample.

As Veale et al. (2016a) pointed out, there is a wide variety between previous inpatient and residential studies regarding admission criteria and sample characteristics. The current sample may be different from some of the previous samples, as there was no fixed admission criteria regarding Y-BOCS severity, whereas studies like Boschen et al. (2010) and Veale et al. (2016a) had an inclusion criterion of Y-BOCS ≥ 30. In addition, the current inpatient service had no criteria regarding the number of previously failed treatments, even though most of the patients had previous treatment attempts for OCD (see **Table 1**). A third distinction is related to economy. The stay at the inpatient unit was financed by the national health care system in Norway (i.e., not self-payed or covered by private insurance providers). Thus, the inpatient service may have been more accessible in Norway as compared to other countries. However, despite these differences, the mean pre-treatment Y-BOCS from the 19 studies described in Veale et al. (2016b) is similar to scores in the current study (see **Figure 2**). The studies with the lowest (Kuelz et al., 2006) and highest (Boschen et al., 2010) mean pre-treatment Y-BOCS in Veale et al. (2016b) are also displayed.

### Comparison of Treatment Completers and Dropouts

As shown in **Table 2**, no significant difference was identified between completers (*n* = 166) and dropouts (*n* = 21) with respect to Y-BOCS, OCI-R, and BDI severity measured at baseline. However, significant differences were found regarding gender, *χ^2^*(1, *N* = 187) = 12.57, *p* = 0.001, and prior inpatient treatment, *χ*^2^(1, *N* = 187) = 6.45, *p* = 0.011. Among dropouts, there was a higher proportion of males and higher percentage participants with previous inpatient treatment.

**Table 2:** Comparison of demographic and diagnostic characteristics among treatment completers and dropouts.

	Completers *(n = 166)*	Dropouts *(n = 21)*		
Characteristic	*%*	*%*	*Phi*	*p*
Female gender	68.1	28.6	0.26	**0.001**
Employed/student	49.3	42.9	0.03	0.711
Married/cohabitant	35.5	28.6	-0.05	0.527
Using antidepressants	53.6	57.1	0.01	0.960
Previous outpatient treatment	99.4	100	0.03	0.721
Previous inpatient treatment	33.5	61.9	0.19	**0.011**

	***M (SD)***	***M (SD)***	***t***	***P***

Age	34.1 (11.4)	35.2 (9.7)	-0.44	0.663
Y-BOCS	25.9 (4.8)	27.5 (4.6)	-1.45	0.149
OCI-R	30.0 (12.7)	32.4 (11.7)	-0.85	0.396
BDI	19.2 (9.9)	19.7 (10.5)	-0.22	0.829

*Significant *p*-values are in boldface. Y-BOCS, Yale-Brown Obsessive-Compulsive Scale; OCI-R, Obsessive-Compulsive Inventory-Revised; BDI, Beck Depression Inventory.*

### Treatment Effectiveness

Assessment, admission, discharge, and follow-up scores for the Y-BOCS, the OCI-R, and the BDI are presented in **Table 3**. Means and standard deviations are reported for each measure for treatment completers and the ITT sample.

**Table 3:** Changes in obsessive-compulsive and depressive symptoms.

Measure	*n*	Assessment *M (SD)*	Admission *M (SD)*	Discharge *M (SD)*	3-month FU *M (SD)*	6-month FU *M (SD)*	Assessment-discharge	Assessment- 6-month FU
							*d*	*d*
Y-BOCS								
Completers	166	25.9 (4.8)	24.6 (5.8)	10.5 (5.2)	14.7 (7.1)	15.1 (7.0)	2.62	1.74
ITT	187	26.0 (4.8)	25.0 (5.8)	12.5 (7.6)	16.2 (8.1)	16.6 (8.0)	1.86	1.40
OCI-R								
Completers	166	30.0 (12.7)	28.4 (12.7)	10.9 (9.4)	15.1 (10.2)	15.6 (10.0)	1.71	1.40
ITT	187	30.2 (12.6)	28.7 (12.5)	13.2 (11.5)	16.9 (11.4)	17.4 (11.1)	1.37	1.18
BDI								
Completers	166	19.2 (9.9)	17.8 (10.1)	7.6 (7.9)	11.2 (9.4)	11.5 (8.8)	1.29	0.87
ITT	187	19.3 (9.9)	18.1 (10.2)	9.1 (9.2)	12.3 (10.0)	12.5 (9.5)	1.06	0.76

*Effect sizes were calculated with Cohens *d*, correcting for related means by using the equation by Morris and DeShon (2002). Y-BOCS, Yale-Brown Obsessive-Compulsive Scale; OCI-R, Obsessive-Compulsive Inventory-Revised; BDI, Beck Depression Inventory; FU, follow-up.*

The results of the repeated measures ANOVA showed a significant main effect of time on Y-BOCS outcome, *F*(2.6,476.2) = 306.0, *p* = 0.001, $\eta_{p}^{2}$ = 0.62. *Post hoc* tests with Bonferroni correction showed a small, but significant decrease in obsessive-compulsive symptoms from assessment phase to treatment start (*p* = 0.014), as well as a significant decrease from treatment start to discharge (*p* = 0.001). The data further indicated an increase in obsessive-compulsive symptoms from discharge to 3-months follow-up (*p* = 0.001), followed by stability in symptom severity between 3-month and 6-month follow-up (*p* = 0.989). On average, this represented a 52% decrease in obsessive-compulsive symptoms from assessment to discharge, and a 36% decrease from assessment to 6-month follow-up.

The analysis was repeated with OCI-R as measure of obsessive-compulsive symptoms. With the exception of a non-significant decrease between assessment phase and treatment start (*p* = 0.061), the same pattern in symptom changes emerged. Overall, there was a statistically significant effect of time on obsessive-compulsive symptoms, *F*(2.2,403.6) = 240.4, *p* = 0.001, $\eta_{p}^{2}$ = 0.68. *Post hoc* tests with Bonferroni correction showed a significant decrease in symptoms between assessment and discharge (*p* = 0.001), a significant increase in symptoms between discharge and 3-month follow-up, (*p* = 0.001), followed by stability in symptom level between 3- and 6-month follow-up (*p* = 0.999). This represented, on average, a decrease of 56% in obsessive-compulsive symptoms from assessment to discharge, and a decrease of 42% from assessment to 6-month follow-up.

Regarding depressive symptoms, the outcome pattern was similar to obsessive-compulsive symptoms. There was a statistically significant effect of time on depressive symptoms, as measured with the BDI, *F*(2.5,465.1) = 112.4, *p* = 0.001, $\eta_{p}^{2}$ = 0.55. *Post hoc* tests with Bonferroni correction showed a non-significant decrease in symptoms between assessment phase and treatment start (*p* = 0.170) and a significant decrease in symptoms between assessment phase and discharge (*p* = 0.001). Furthermore, there was a significant increase in symptoms between discharge and 3-month follow-up (*p* = 0.001), followed by stability in symptom level between 3- and 6-month follow-up (*p* = 0.999). On average, this represented a 53% decrease in depressive symptoms from assessment to discharge, and a 35% decrease from assessment to 6-month follow-up.

**FIGURE 3:**
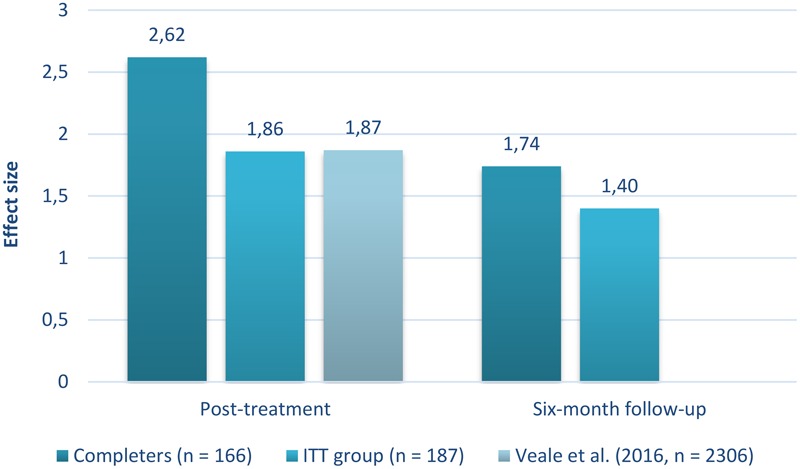
Comparison of Y-BOCS effect sizes between studies on inpatient and residential treatment for OCD. The Y-BOCS effect size estimates from the current study were compared to the Y-BOCS effect size estimate in the review study of 19 inpatient and residential treatment programs using CBT for OCD (Veale et al., 2016b). The current study had a fixed duration of 3 weeks, whereas the mean treatment duration in Veale et al. (2016b) was 10.4 weeks. Y-BOCS, Yale-Brown Obsessive-Compulsive Scale; ITT group, intention-to-treat sample.

The within-subject effect-sizes for changes in obsessive-compulsive and depressive symptoms are presented in **Table 3**. The effect sizes from assessment phase to discharge, as well as assessment phase to follow-up, were large for obsessive-compulsive symptoms and moderate to large for depressive symptoms. See **Figure 3** for a comparison of effect size estimates from the current study and the meta-analysis of 19 inpatient and residential treatment programs using CBT for OCD (Veale et al., 2016b). The comparison showed that the ITT group’s effect size from assessment phase to discharge was similar to the effect size reported in Veale et al. (2016b), whereas the treatment completers had a higher effect size estimate.

### Clinically Significant Change

Following Mataix-Cols et al.’s (2016) criteria, the Y-BOCS results for the ITT group at post-treatment were as follows: 79.7% were classified as treatment responders, 5.3% as partial responders, 57.8% met criteria for remission, whereas 15% had no change in symptoms. At 6-month follow-up, 61.5% were classified as treatment responders and 8.0% as partial responders. A proportion of 34.2% met criteria for remission, whereas 30.5% had no change in symptoms.

Variability in how treatment response was defined did lead to different estimates of treatment efficacy and relapse rates. Following Fisher and Wells’ (2005) clinically significant change criteria for Y-BOCS scores, 71.1% of treatment completers and 63.1% of the ITT group were classified as recovered at post-treatment. Similar results were found for OCI-R scores; 65.6% of treatment completers and 58.2% of the ITT sample met criteria for recovery at discharge. At 6-month follow-up, 35.3 – 40.1% of the ITT sample were classified as recovered, whereas an additional proportion of 10.7 – 12.8% were classified as significantly improved, as measured by OCI-R and Y-BOCS. See **Table 4** for a summary of the results from the clinically significant change analyses.

**Table 4:** Clinically significant change in obsessive-compulsive symptoms.

Measure	Treatment response (Recovered)	Partial response (Improved)	Remission (≤cut-off)	No change
***Post-treatment***				
Y-BOCS				
Completers	89.9 (71.1)	5.4 (13.3)	65.0 (77.7)	4.8 (15.7)
ITT	79.7 (63.1)	5.3 (11.8)	57.8 (69.0)	15.0 (25.1)
OCI-R				
Completers	65.6	14.1	85.9	20.2
ITT	58.2	12.5	78.3	29.3
***Six-month follow-up***				
Y-BOCS				
Completers	69.3 (39.8)	8.4 (14.5)	38.6 (48.2)	22.3 (45.8)
ITT	61.5 (35.3)	8.0 (12.8)	34.2 (42.8)	30.5 (51.9)
OCI-R				
Completers	45.2	11.4	75.9	43.4
ITT	40.1	10.7	69.5	49.2

*Results are displayed in percentages. For Y-BOCS, Mataix-Cols et al.’s (2016) criteria are reported as the primary clinically significant change estimate, whereas the criteria recommended by Fisher and Wells (2005) are included as an additional change measure. The criteria by Fisher and Wells (2005) are listed in parentheses. For OCI-R, similar criteria as used by Solem et al. (2009) were applied. Y-BOCS, Yale-Brown Obsessive-Compulsive Scale; OCI-R, Obsessive-Compulsive Inventory-Revised; ITT, intent-to-treat sample.*

### Influence of Antidepressant Use

At baseline, 54% of the sample reported use of antidepressant medication (mainly SSRI’s). The most frequently used were Cipralex (*n* = 29) and Zoloft (*n* = 21), followed by Anafranil, Seroxat, and Fluoxetin (each with *n* = 7). Regarding dose, 60% were higher than the drug’s DDD, 35% were at the level of the recommended DDD, whereas 5% were below the DDD. A proportion of 76% held dose levels unchanged during the whole treatment phase. Before treatment start, 4% reduced the dose and 9% stopped using anti-depressants. Between post-treatment and 6 months follow-up, 1% reduced the dose and 11% stopped using antidepressants.

No significant difference was identified between antidepressant users and non-users with respect to Y-BOCS post-treatment score [*M* = 12.88, *SD* = 7.32; *M* = 12.12, *SD* = 7.95, *t*(185) = 0.67, *p* = 0.501] and Y-BOCS 6-month score [*M* = 16.62, *SD* = 7.68; *M* = 16.53, *SD* = 8.29, *t*(185) = 0.08, *p* = 0.940]. In addition, there was no relationship between dose and Y-BOCS treatment outcome, neither at post-treatment, *r*(82) = 0.09, *p* = 0.413, nor 6-month follow-up, *r*(82) = 0.06, *p* = 0.589.

### Predictors of Relapse

A logistic regression analysis was used to explore predictors of relapse at 6-month follow-up. Patients who relapsed following discharge comprised 28.9% of the sample (*n* = 54). Pre-treatment levels of obsessive-compulsive symptoms (Y-BOCS) emerged as the only significant predictor of relapse, *B* = 0.17, *SE* = 0.06, *p* = 0.003. Each unit increase in Y-BOCS severity at pre-treatment was associated with an increase in the odds of relapse by a factor of 1.19 (95% CI: 1.06 – 1.33). Neither gender (*B* = 0.44, *SE* = 0.46, *p* = 0.347), age (*B* = -0.02, *SE* = 0.02, *p* = 0.262), marital status (*B* = 0.36, *SE* = 0.47, *p* = 0.443), pre-treatment levels of depression (*B* = -0.01, *SE* = 0.02, *p* = 0.624), nor attendance to the 3-day follow-up stay 3 months after discharge (*B* = 0.31, *SE* = 0.52, *p* = 0.546) emerged as significant predictors of relapse.

## Discussion

This is the first study investigating the long-term outcome of a 3-week inpatient treatment for OCD. Consistent with this study’s hypothesis, the results indicated that the treatment delivered was effective. At discharge, 79.7% of the patients were classified as treatment responders regarding obsessive-compulsive symptoms. The dropout rate was low (11.2%), indicating that the treatment was considered acceptable for most patients. There was an increase in obsessive-compulsive symptoms from post-treatment to 3-month follow-up, followed by stability in symptom severity between 3 and 6 months. At 6-month follow-up, 61.5% of the patients showed a full treatment response, indicating that some patients were still unchanged or had relapsed. The outcome pattern of depressive symptoms was similar to obsessive-compulsive symptoms. Antidepressant use appeared not to influence the outcome. Only pre-treatment level of obsessive-compulsive symptoms emerged as a significant predictor of relapse.

Compared to previous studies of inpatient and residential treatment for OCD (e.g., Kordon et al., 2005; Stewart et al., 2005; Veale et al., 2016a), where the majority report treatment durations of 2–3 months, the current study displays a similar treatment effect at discharge. In the meta-analysis of residential and inpatient OCD treatment, Veale et al. (2016b) found a mean reduction in Y-BOCS scores from baseline to post-treatment at 10.7 points, whereas the mean reduction score in the ITT-sample in our study was 12.5. This indicates that a brief inpatient format can produce a similar post-treatment result as inpatient formats of longer duration.

Despite very encouraging post-treatment results, there was some relapse at follow-up. Overall, the current changes in obsessive-compulsive symptoms from assessment phase to 6-month follow-up showed large effect sizes, but the results indicated an increase in obsessive-compulsive symptoms from post-treatment to 3-month follow-up, followed by stability in symptom severity between 3-month and 6-month follow-up. The results of previous inpatient and residential studies reporting long-term outcomes are inconsistent, with some studies reporting the gains to be stable (e.g., Kordon et al., 2005; Rufer et al., 2005; Stewart et al., 2009), whereas some found a slight deterioration (McKenzie and Marks, 2003; Veale et al., 2016a). One explanation for the differential results may be methodological, as the heterogeneity in treatment and populations among the studies who report follow-up data is large. With the exception of Veale et al. (2016a, *N* = 124 at Y-BOCS at 6–12 months follow-up), most of the previous inpatient and residential treatment studies reporting follow-up data had small sample sizes (range of *N*: 16 – 44) and/or low response rates. In comparison, a strength of our study is a sample size of 142 at 3-month follow-up (i.e., 86% of treatment completers) and 104 at 6-month follow-up (i.e., 63% of treatment completers).

Furthermore, the different symptom trends at follow-up between studies might be explained by differences in treatment duration. The increase in symptoms at 3-month follow-up is in line with the meta-analytic findings of Jónsson et al. (2015), suggesting that there could be more relapse in patients receiving intensive treatment. Jónsson et al. (2015) compared treatment effects of intensive outpatient CBT with standard weekly CBT. They found a significantly larger post-treatment effect in the intensive treatment condition, but this difference was no longer present at 3-month follow-up. According to Jónsson et al. (2015), this was mainly due to some deterioration among patients in the intensive CBT-formats. The higher relapse rate in intensive treatment conditions may be due to fewer opportunities to consolidate and generalize the learning to the patients’ home environment. Craske et al. (2008) recommend spacing of exposure trials, as well as a lot of variability throughout exposure, to enhance the accessibility and retrievability of learning. In our study, the majority of patients were not settled near-by the inpatient unit. The leave home (one weekend during the treatment phase) was designed to address the issues of variability and generalizing learning, but one weekend may have been too short to consolidate this learning sufficiently.

In general, there is a need for more research to clarify the relative long-term effectiveness of intensive versus standard treatment formats in both inpatient and outpatient treatment settings, as well as further research into how to maintain the good post-treatment effects of the brief and intensive format. Use of controlled trials with large samples yielding high response rates at follow-up are recommended.

As hypothesized, depressive symptoms demonstrated a similar outcome pattern as obsessive-compulsive symptoms. There was a significant decrease in symptoms between pre-treatment and post-treatment, an increase between post-treatment and 3-month follow-up, followed by stability in symptoms between 3-month and 6-month follow-up. Overall, this resulted in a 35% decrease in depressive symptoms from pre-treatment to follow-up. These results are in line with previous studies examining depressive symptoms during inpatient and residential treatment for OCD (e.g., Stewart et al., 2009; Veale et al., 2016a). As Stewart et al. (2009) also pointed out - whether the improvement in depressive symptoms is primary or secondary to reduction in obsessive-compulsive symptoms, or whether the change occurred in both symptom types simultaneously, is unknown. Nevertheless, improvement from depressive symptoms is of clinical relevance, as comorbid depression severity has been found to contribute to poor quality of life (e.g., Masellis et al., 2003) and occupational disability (e.g., Mancebo et al., 2008).

Approximately half of the sample used antidepressant medication during treatment, but antidepressant use appeared not to influence the outcome. There was no significant difference in Y-BOCS post-treatment and 6-month score between antidepressant users and non-users. Also, there was no relationship between dose and Y-BOCS treatment outcome. These results are in line with previous studies examining the influence of antidepressant medication on OCD inpatient treatment outcome (e.g., Kordon et al., 2005; Rufer et al., 2005; Langner et al., 2009). In a 2-year follow-up study of 74 inpatients with OCD, Kordon et al. (2005) found CBT and combined therapy (CBT + serotonin-reuptake inhibitors treatment) to be equally effective both at post-treatment and follow-up. Furthermore, no differences in the obsessive-compulsive scores were observed between those who continued antidepressant medication and those who discontinued.

Only pre-treatment level of obsessive-compulsive symptoms emerged as a significant predictor of relapse. Neither gender, age, marital status, pre-treatment levels of depression, nor attendance to the follow-up stay 3 months after discharge emerged as significant predictors of relapse. The results are not in accordance with the results of Stewart et al. (2009), where patients who relapsed were found to be significantly more likely to be living alone. However, the results are in line with Knopp et al. (2013) systematic review of predictors of response to psychological therapies in OCD, as they found OCD symptom severity as one of the few variables that was relatively consistently related to outcome.

### Limitations

Due to the naturalistic design of the study, there were no independent raters of diagnostic information at baseline, and there was no control group. The lack of control group may give a false impression of efficiency. However, spontaneous recovery from OCD is rare. In addition, as the inpatient treatment program was not strictly manualized, there is no consistent information about the relative contribution of cognitive and metacognitive elements added to ERP.

Inevitably, data from large service evaluations are likely to have missing data, which can bias the effect of treatment on response. The analysis adjusted for missing data at follow-up by using EM and LOCF as imputation strategies and therefore has to be interpreted with caution. Furthermore, there was variability in the measures used, as both the self-report and interview versions of the Y-BOCS were used. Similarly, for ratings of depressive symptoms, the first included patients used BDI, while the others used BDI-II. However, the BDI and the BDI-II are strongly correlated (Dozois et al., 1998), as are Y-BOCS and Y-BOCS-SR (Steketee et al., 1996). In the current study, there were no significant differences in baseline scores between Y-BOCS and Y-BOCS-SR or between BDI and BDI-II.

Another limitation concerns follow-up assessment. The diagnostic status of OCD was not re-evaluated. Inclusion of other potentially clinically important indicators of treatment outcome such as anxiety and functional outcomes across multiple settings (e.g., home, work, social life) would have improved the study. Given that OCD is associated with impaired quality of life and functioning (Asnaani et al., 2017), inclusion of an inventory such as EuroQol (EuroQol-Group, 1990) could have brought information regarding how changes in OCD severity are associated with changes in functioning and quality of life. Measurement of the use of outpatient care after discharge, as well as an analysis of the cost-effectiveness of the inpatient treatment, would also have improved the study. Finally, what happens with their obsessive-compulsive symptoms after 6 months remains unknown.

## Conclusion

The current study’s inpatient unit for OCD was established in 2008. At the time, evidence-based treatments of OCD (i.e., ERP and CBT) were difficult to get in Norway due to few available ERP specialists. Therefore, the 3-week inpatient program was originally established as a practical arrangement to handle referrals from the whole country. However, there was an explicit wish to establish local specialized OCD-teams that could carry through the OCD-treatment closer to people’s homes and in a more cost-effective way. This resulted in the national implementation of OCD treatment in Norway and the establishment of 30 specialized OCD-teams (Kvale and Hansen, 2014).

As far as we know, this is the first study to look at the effectiveness of a brief (3 weeks) version of inpatient treatment in such a large sample size, combined with long-term follow-up data with good response rates. This study replicates previous findings concerning inpatient and residential treatment being an effective treatment format (e.g., Stewart et al., 2005, 2009; Veale et al., 2016a) for severe OCD, and extends these results to a brief version of inpatient treatment. However, the increase in symptoms between post-treatment and 3-month follow-up underlines the need for further research into predictors of relapse in inpatient and residential treatment, as well as further research into how to maintain the good post-treatment effects of the brief and intensive format. One possibility may be booster sessions delivered by video chatting applications (Vogel et al., 2014).

## Author Contributions

BH and IG contributed to conception and design of the study. TG was responsible for the data collection, data analysis, interpretation, drafting, and revising the work. SS participated in designing the project, data analysis and interpretation, as well as revising the manuscript. TG wrote the first draft of the manuscript, whereas all authors contributed to manuscript version, read and approved the submitted version.

## Conflict of Interest Statement

The authors declare that the research was conducted in the absence of any commercial or financial relationships that could be construed as a potential conflict of interest.

## Footnotes

1 The calculation was conducted by the software program “Comprehensive meta-analysis,” where the mean, SD, and number of participants were taken into consideration.
